# Comparing the Metabolic Profile of Patients Affected by Acute-Onset Neuropsychiatric Syndrome PANS and Tourette Syndrome: Preliminary Data

**DOI:** 10.3390/medsci14020232

**Published:** 2026-05-01

**Authors:** Federica Murgia, Antonio Noto, Marcello Giuseppe Tanca, Carola Costanza, Valeria Marletta, Sara Carucci, Antonella Gagliano, Luigi Atzori

**Affiliations:** 1 Clinical Pathology and Microbiology Laboratory, SS Trinità Hospital, ASL, 09121 Cagliari, Italy; 2 Clinical Metabolomics Unit, Department of Biomedical Sciences, University of Cagliari, 09042 Cagliari, Italy; latzori@unica.it; 3 Child and Adolescent Neuropsychiatry Unit, Department of Medical Sciences and Public Health, University of Cagliari and “A Cao” Pediatric Hospital, 09121 Cagliari, Italy; 4 Department of Neuroscience, Oasi Research Institute-IRCCS, 94018 Troina, Italy; 5 Child and Adolescent Neuropsychiatry, Department of Medicine and Surgery, “Kore” University of Enna, 94100 Enna, Italy

**Keywords:** Tourette syndrome, pediatric acute-onset neuropsychiatric syndrome, metabolomics, biomarkers’ evaluation

## Abstract

**Background**: Pediatric Acute-Onset Neuropsychiatric Syndrome (PANS) shares numerous clinical features with Tourette syndrome (TS), notably the presence of tics and frequent comorbidities such as obsessive-compulsive disorder, irritability, and ADHD-like behaviors, often indistinguishable, particularly in the early stages of the two syndromes. Also, pathogenic similarities between PANS and TS constitute a diagnostic challenge, highlighting the need for biomarkers elucidating the underpinnings of the two disorders. In this context, metabolomics has emerged as a powerful tool for identifying distinct biochemical patterns in various diseases. We previously compared PANS, autism patients, and controls, identifying specific metabolic patterns. However, no studies have directly compared the metabolomic profiles of Tourette syndrome and PANS patients. The present study aims to compare the serum metabolomic profiles of TS patients with those of PANS and healthy controls to advance the molecular understanding and clinical differentiation of these two pediatric neuropsychiatric disorders. **Methods:** Thirty-four PANS patients and twenty-three Tourette patients were matched with twenty-five healthy subjects (C), and their blood samples were analyzed with ^1^H NMR spectroscopy. Subsequently, data were analysed with multivariate and univariate statistical approaches. **Results:** Supervised models indicated that the metabolomic profile of TS patients was significantly different from that of controls (*p* = 0.02), with altered concentrations of glutamate, glycerol, glycine, lactate, and proline. No significant differences were found in the comparison between PANS and TS patients. **Conclusions**: These preliminary data suggest that Tourette and Pans also seem to share the metabolic profiles, while differences were found in TS patients compared to controls. On the other hand, the PANS phenotype comprises symptoms that largely overlap with those of all other NDDs, including TS, outlining a spectrum of disorders that share common pathogenetic pathways. Larger studies are needed to confirm these findings.

## 1. Introduction

Pediatric Acute-Onset Neuropsychiatric Syndrome (PANS) is defined by the abrupt onset of obsessive–compulsive symptoms and/or severe food restriction, accompanied by a constellation of behavioral and neurological manifestations that often require a multidisciplinary clinical approach [1,2]. The diagnosis requires the presence of at least two concurrent neuropsychiatric symptoms such as anxiety, emotional lability and/or depression, irritability/aggression and/or oppositional behaviors, behavioral regression, deterioration in school performance, sensory or motor abnormalities, or somatic signs and symptoms beyond the core criteria [2].

This syndrome is frequently triggered by infections, with a range of potential pathogens implicated, not limited to Group A Streptococcus, which is specifically associated with Pediatric Autoimmune Neuropsychiatric Disorders Associated with Streptococcal Infections (PANDAS) [3]. Building on the PANDAS framework, diagnostic criteria were revised and broadened, leading to the formulation of the PANS entity [2].

PANS shares numerous clinical features with Tourette syndrome (TS), notably the presence of tics and frequent comorbidities such as obsessive–compulsive symptoms, irritability, and ADHD-like behaviors—which are often indistinguishable from those seen in TS, particularly in their early stages [2,4].

TS is a childhood-onset neurodevelopmental disorder characterized by motor and vocal tics that follow a fluctuating course and persist for more than one year without a tic-free interval exceeding three consecutive months [5].

Although its pathophysiology remains incompletely understood, TS is thought to arise from a complex interaction of genetic predisposition, dopaminergic neurotransmission abnormalities, and immune–inflammatory mechanisms [6,7]. Moreover, increasing evidence supports immune-mediated mechanisms and inflammatory conditions relevant in TS as well as in obsessive–compulsive and related disorders [8,9]. The clinical and pathogenic overlap between PANS and TS represents a significant diagnostic challenge and highlights the need for objective biomarkers to aid in differential diagnosis and support treatment strategies [10]. Preclinical studies in animals have highlighted that autoimmune responses triggered by streptococcal infections may play a role in the pathogenesis of Tourette syndrome, supporting the hypothesis of immune-mediated mechanisms underlying tic disorders [11]. Furthermore, a shared immuno-inflammatory basis across PANS, TS, and related neuropsychiatric conditions has been proposed, reinforcing the rationale for exploring molecular biomarkers [12].

Systemic immune activation, oxidative stress, and metabolic adaptation are known to influence amino acid metabolism, mitochondrial function, nitrogen handling, and redox balance. These processes can generate measurable alterations in circulating metabolites, making peripheral biofluids such as serum a relevant matrix for capturing disease-related biochemical signatures.

In this context, metabolomics, which enables the quantitative analysis of small endogenous metabolites in biological fluids, has emerged as a powerful tool for identifying distinct biochemical patterns in various diseases, including neuropsychiatric disorders [13,14].

We previously applied ^1^H NMR spectroscopy to PANS and other neurodevelopmental conditions, identifying specific metabolic patterns in PANS patients, including altered levels of tryptophan, glycine, and glutamine, as well as markers of oxidative stress and neuroinflammation [15,16].

However, to date, no studies have directly compared the metabolomic profiles of Tourette syndrome and PANS patients. Given the clinical and pathogenic similarities, it remains unclear whether these conditions are characterized by distinct metabolic signatures or partially shared biochemical alterations that could serve as diagnostic biomarkers.

In this exploratory study, we hypothesized that TS and PANS may exhibit partially overlapping but not fully identical metabolomic profiles, reflecting both shared and condition-specific biological processes.

The present study aims to provide preliminary metabolomics data on the serum metabolomic profile of patients with Tourette syndrome using ^1^H NMR spectroscopy and to compare these findings with those of children diagnosed with PANS and healthy controls. The objective was to identify discriminating metabolites and altered metabolic pathways, thereby contributing to the molecular characterization and potential clinical differentiation of these pediatric neuropsychiatric disorders.

## 2. Materials and Methods

### 2.1. Study Design

Serum samples were collected from individuals diagnosed with PANS or Tourette syndrome, as well as from healthy controls. The analytical workflow included an initial comparison of the hydrophilic metabolic profiles of Tourette patients, encompassing amino acids, sugars, biogenic amines, fatty acids, and organic acids, with those of the control group to identify metabolic alterations associated with the disorder. Subsequently, we examined the metabolic patterns of Tourette and PANS patients to identify shared and unique features that could help distinguish the two conditions.

This study followed the principles of the Declaration of Helsinki and received approval from the Ethics Committee of Cagliari University Hospital (ethical approval number: Prot. PG/2019/7413; date in the Institutional Review Board Statement: 29 May 2019) [17]. Written informed consent for participation and data publication was obtained from parents or legal guardians of all enrolled minors. Recruitment of both patients and controls took place between June 2019 and May 2020 at the outpatient service of the Child and Adolescent Neuropsychiatry Unit at the “G. Brotzu” Hospital Trust, Cagliari. PANS diagnoses were confirmed by two child psychiatrists using the 2010 NIMH criteria. Tourette diagnoses were made according to the Diagnostic and Statistical Manual of Mental Disorders (DSM-5^®^) [18].

A total sample of 33 PANS patients and 26 Tourette patients were recruited. The healthy control (HC) group consisted of 28 neurotypical children from the same geographical area, matched for age and sex. Age distribution across groups was evaluated and considered in the statistical interpretation, given the known developmental sensitivity of pediatric metabolomic profiles. Medication exposure in sampling was controlled by making sure that all participants had undergone a drug-free period of at least 6 weeks. The interruption of nutraceutical supplements at least 6 weeks before admission to this study was also requested. Disease duration was variable, ranging from 1 to 36 months. Nevertheless, at the time of clinical assessment, all recruited clinical subjects (both PANS and TS) showed active psychiatric symptoms (onset or waxing phase of a relapsing–remitting course). A large proportion of subjects with PANS (26/33; 78.7%) had had a recent infectious episode or had a history of recurrent infectious episodes. None of them had acute symptoms of microbial infection at the time of assessment. Among TS subjects, 20/26 (76.9%) had comorbid attention-deficit/hyperactivity disorder symptoms and/or specific learning or motor coordination difficulties. None of them had intellectual disability or autism spectrum disorder.

All participants underwent laboratory testing, including complete blood counts, renal and liver function tests, a mineral panel, thyroid markers, and inflammatory indices to rule out metabolic or systemic diseases. Moreover, all enrolled patients received physical, neurological, and psychiatric assessments and were evaluated using a comprehensive set of standardized scales and questionnaires to assess symptoms and clinical severity. These included the Pediatric Anxiety Rating Scale (PARS), the Pediatric Acute Neuropsychiatric Symptom Scale (PANSS), the Children’s Yale–Brown Obsessive Compulsive Scale (CY-BOCS), the Yale Global Tic Severity Scale (YGTSS), the Children’s Global Assessment Scale (C-GAS), the Universidade Federal de Minas Gerais Sydenham’s Chorea Rating Scale (USCRS), and the Full-Scale Intelligence Quotient (FSIQ), assessed with the Wechsler Intelligence Scale for Children, Fourth Edition (WISC-IV). Exclusion criteria were as follows: (I) autoimmune disorders or cancer; (II) other medical, neurological, or psychiatric conditions; (III) ongoing treatment with corticosteroids or non-steroidal anti-inflammatory drugs; and (IV) absence of written informed consent from parents or legal guardians, or withdrawal of consent by the patients.

### 2.2. Sample Preparation and Data Analysis

Blood samples (10 mL, collected via venipuncture) from 33 PANS patients, 26 Tourette patients, and 28 healthy controls (C) were obtained after an overnight fast (12 h) and centrifuged at 2500× *g* for 10 min at 4 °C. Sera were stored at −80 °C until analysis and processed as described in our previous study.

Briefly, samples were thawed and subjected to the Folch method to extract and separate hydrophilic and lipophilic metabolites. Aliquots (10 µL) from each sample were combined to generate pooled quality control (QC) samples. QC samples were analyzed at the beginning and end of the sequence and used to assess instrumental stability. Their behavior was further evaluated through PCA clustering to verify analytical reproducibility. For extraction, 400 µL of each serum sample was mixed with 600 µL of methanol, 600 µL of chloroform, and 175 µL of Milli-Q water. Samples were vortexed for 1 min and centrifuged for 30 min at 1700 *g* at room temperature. After Folch extraction, 700 µL of the hydrophilic phase containing low-molecular-weight metabolites (amino acids, sugars, etc.) was concentrated overnight using a speed vacuum centrifuge.

For the ^1^H NMR analysis, the concentrated aqueous phase was resuspended in 690 µL of D_2_O phosphate buffer (pH 7.4) and 10 µL of 5.07 mM trimethylsilyl propionic acid (TSP). TSP was added as an internal reference for chemical shifts (0 ppm). A total of 650 µL of the solution was transferred into a 5 mm NMR tube. Samples were analyzed using a Varian UNITY INOVA 500 spectrometer (Agilent Technologies, Santa Clara, CA, USA), operating at 499 MHz and equipped with a 5 mm triple-resonance probe with *z*-axis pulsed field gradients and an autosampler with 50 positions. One-dimensional ^1^H NMR spectra were acquired at 300 K using a pre-saturation pulse sequence to suppress the residual water signal. Spectra were collected with a spectral width of 6000 Hz, a frequency of 2 Hz, an acquisition time of 1.5 s, a relaxation delay of 2 ms, and a 90° pulse of 9.5 μs. A total of 256 scans were acquired. Each Free Induction Decay (FID) was zero-filled to 64k points and multiplied by a 0.5 Hz exponential line-broadening function.

After acquisition, spectra were manually processed (phase and baseline correction) using MestReNova software (version 8.1, Mestrelab Research S.L.). Each NMR spectrum was then divided into consecutive bins of 0.04 ppm. The analyzed spectral region ranged from 0.6 to 8.6 ppm. The regions between 4.60–5.20 ppm and 5.24–6.60 ppm were excluded to remove variability due to residual water resonance and noisy regions. The integrated area of each bin was normalized to a constant sum of 100. This normalization approach was selected to reduce inter-sample variability and account for differences in total metabolite concentration. The final dataset consisted of a 150 × 86 matrix, with columns representing normalized bin areas (variables) and rows representing samples (subjects).

### 2.3. Statistical Analysis

Multivariate statistical analyses were performed on the NMR matrix using SIMCA-P software (version 16.0, Sartorius Stedim Biotech, Umeå, Sweden). Variables were Pareto-scaled: Pareto scaling was applied to balance the contribution of high- and low-intensity variables while preserving data structure. Initial unsupervised analyses were carried out using principal component analysis (PCA) to explore intrinsic clustering and identify outliers. Hotelling’s T^2^ test was applied for this purpose. QC samples were included in the PCA to assess clustering and confirm analytical stability.

Supervised models were subsequently constructed, specifically Partial Least Squares Discriminant Analysis (PLS-DA) and Orthogonal Partial Least Squares Discriminant Analysis (OPLS-DA). These models maximize discrimination among predefined classes and were used to distinguish PANS, Tourette syndrome, and healthy subjects. Model strength was evaluated using variance and predictive ability parameters (R^2^X, R^2^Y, and Q^2^). Given the known risk of overfitting in supervised models applied to relatively small cohorts, model interpretation was performed cautiously. Additionally, permutation testing (n = 400) was performed to validate each model by comparing its performance with that of randomly permuted datasets. Cross-validated ANOVA (CV-ANOVA) was also applied to assess statistical significance between patient groups (*p* < 0.05).

Variables with VIP (Variable Importance in Projection) values > 1 in the supervised models, along with their associated loading plots, were identified as the most influential metabolites. Metabolite identification from binned spectra was achieved by combining bin-based statistical outputs with spectral annotation using Chenomx NMR Suite and reference databases, allowing assignment of biologically relevant metabolites associated with discriminant bins. Identified and quantified metabolites were annotated using Chenomx NMR Suite 7.1 (Chenomx Inc., Edmonton, AB, Canada).

GraphPad Prism software (version 7.01; GraphPad Software, San Diego, CA, USA) was used for univariate statistical analyses. The significance of metabolites differentiating patient classes was evaluated using the Mann–Whitney U test, followed by ROC curve analysis to assess sensitivity and specificity (*p* < 0.05). No correction for multiple comparisons was applied; therefore, results should be interpreted as exploratory and potentially affected by type I error inflation. Metabolic pathway analysis was performed using MetaboAnalyst 5.0, a web-based platform for visualization and biological interpretation [www.metaboanalyst.ca accessed on 1 April 2026].

## 3. Results

A total of 34 PANS patients, 23 Tourette outpatient patients, and 25 controls were recruited. Demographic details are reported in Table 1. In particular, the number of subjects per class, the number of females and males, and their ages are reported.

Data from the psychodiagnostics scales (PARS, PANSS, CYBOCS, YGTSS, C-GAS, WISC-IV, and USCRS) used for the clinical evaluation of the enrolled patients are reported in Table 2.

The ^1^H NMR analysis enabled the identification of 44 hydrophilic metabolites, including amino acids, fatty acids, sugars, and biogenic amines.

Initially, principal component analysis (PCA) was conducted on the complete binned dataset comprising Tourette, PANS, and control samples. Hotelling’s T^2^ test revealed one strong outlier within the PANS group, which was subsequently excluded from further analyses.

**Figure 1:**
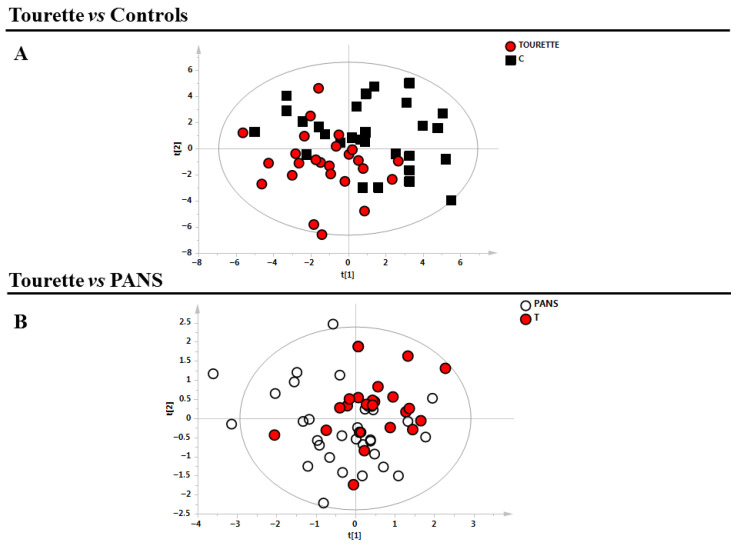
Supervised models of the analysed classes. (**A**) Tourette patients (n = 23, red circles) vs. control subjects (n = 25, black squares) (**B**) PANS (n = 34, white circles) vs. Tourette patients (red circles). PANS = Pediatric Acute-Onset Neuropsychiatric Syndrome.

Supervised models (PLS-DA) were then constructed to compare Tourette (T) versus healthy controls (C), followed by PANS (P) versus Tourette (Figure 1).

Clear separation of samples, in line with their clinical classifications, was observed only for the comparison between T and C, while no differences were observed in the metabolic profile of T and P. All models were validated using the corresponding permutation tests (Table 2). Moreover, we performed PLS correlation analysis between clinical parameters and the metabolic profile of Tourette patients. The values of the relative R^2^ are reported in Table 3.

The most significant variables for the model (T vs. C) were identified using loadings plot analysis along with their associated VIP scores. Metabolites with VIP values greater than 1 were selected and subsequently examined through univariate testing using the Mann–Whitney U test.

**Figure 2:**
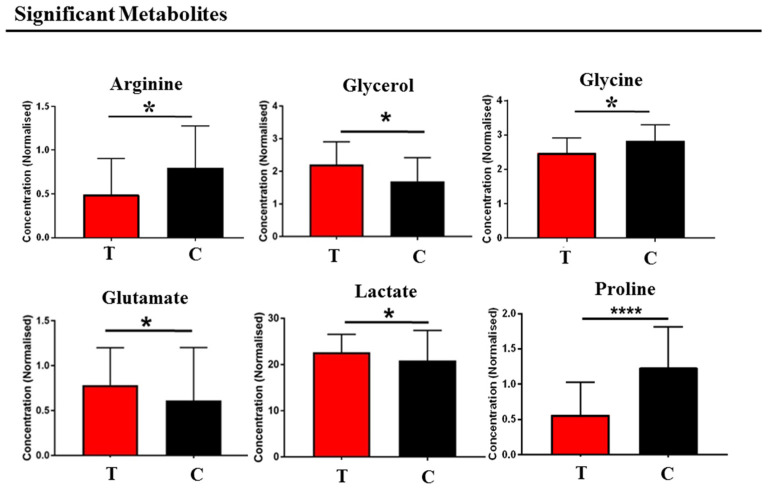
Comparison between Tourette and healthy subjects. Bar graphs of the metabolites exhibit a *p*-value of <0.05 (Mann–Whitney U-test). Red bars represent the Tourette class, and black bars represent the control class. PANS = Pediatric Acute-Onset Neuropsychiatric Syndrome. Error bars represent the standard deviation of each distribution. * *p* < 0.05, and **** *p*-value < 0.0001.

Applying a significance threshold of *p* < 0.05, the metabolites showing the most pronounced differences between Tourette patients and healthy controls were arginine, glycerol, glycine, glutamate, lactate, and proline (Figure 2).

**Figure 3:**
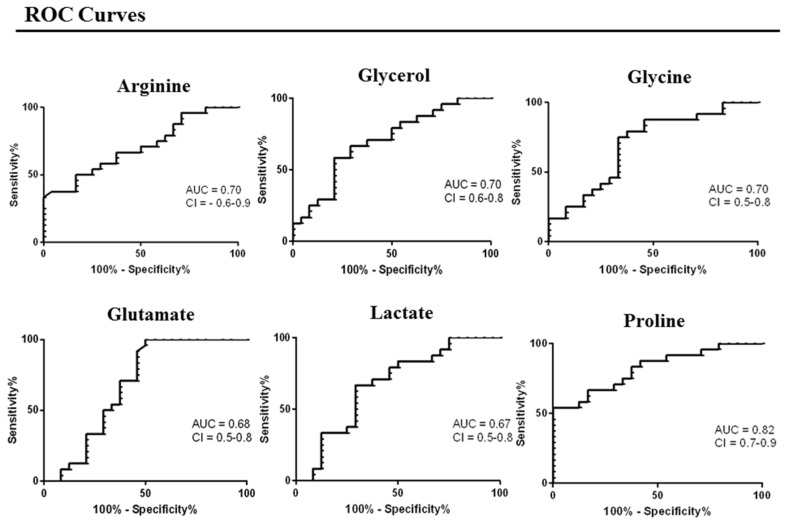
Receiver operating characteristic (ROC) curve analysis of the significant metabolites: arginine, glycerol, glycine, glutamate, lactate, and proline.

**Figure 4:**
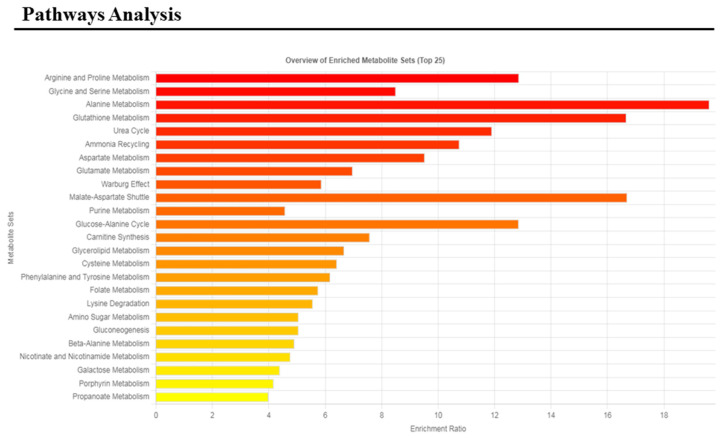
Metabolic pathway analysis of the comparison between control subjects and Tourette patients.

Subsequently, these metabolites were used for receiver operating characteristic (ROC) curve analysis (Figure 3) and for pathways analysis (Figure 4).

The altered pathways with *p*-values < 0.05 were arginine and proline metabolism, glycine and serine metabolism, alanine metabolism, glutathione metabolism, urea cycle, ammonia recycling, and aspartate and glutamate metabolism.

## 4. Discussion

The present serum ^1^H NMR analysis identified a coherent biochemical fingerprint that differentiated Tourette syndrome from neurotypical controls, whereas no statistically supported separation was observed between Tourette syndrome and Pediatric Acute-Onset Neuropsychiatric Syndrome. This pattern is compatible with clinical overlap between these conditions and with convergent models implicating cortico-striato-thalamo-cortical circuit dysfunction modulated by neuroimmune signaling and systemic metabolic adaptation [6,7,12].

Across multivariate and univariate approaches, the most informative metabolites comprised arginine, proline, glycine, glutamate, lactate, and glycerol, and pathway enrichment implicated arginine and proline metabolism, glycine and serine metabolism, glutathione metabolism, the urea cycle and ammonia recycling, and aspartate and glutamate metabolism. These pathways converge on mitochondrial redox regulation and anaplerotic support of the tricarboxylic acid cycle, providing a physiologically plausible framework linking peripheral organ homeostasis with processes that may be relevant to brain circuit function. When contextualized against recent metabolomics work, the present findings reinforce the recurrent implication of amino acid and nitrogen handling in Tourette syndrome while highlighting between-study heterogeneity. In a combined untargeted and targeted plasma metabolomics study, Xi et al. proposed candidate biomarkers including L-arginine along with metabolites linked to the ornithine–proline and glutamatergic pathways (L-glutamate, L-ornithine, D-proline, and D-pipecolic acid) [19]. A recent systematic review and meta-analysis also highlighted altered glutamic acid as one of the more consistently reported biomarkers across studies [20]. The overlap at the pathway level supports biological consistency despite differences in analytical platforms, sampling conditions, age distributions, and medication exposure.

Within the arginine–proline module, the relative perturbations are consistent with immune-mediated modulation of nitrogen and redox metabolism, where inflammatory cell activity may consume arginine via iNOS or arginase pathways, influencing systemic nitrogen flux and redox balance, which may indirectly impact neuronal function. The reduction in circulating arginine observed here can be compatible with an immune–metabolic model in which arginine availability is constrained by increased utilization in activated myeloid cells via inducible nitric oxide synthase or by increased arginase activity, both of which can be engaged during low-grade inflammation and can influence neuronal function through nitric oxide signaling, vascular tone, and redox balance [7,10]. Proline is metabolically coupled to glutamate and ornithine through the proline–pyrroline–5-carboxylate cycle and can modulate mitochondrial reactive oxygen species production and NAD(P)H availability [21]. Perturbation of this axis provides a mechanistic link between nitrogen flux, redox homeostasis, and neurotransmitter precursor availability.

Enrichment of glutathione- and ammonia-related pathways further supports a redox and nitrogen-stress component in the biochemical phenotype. Oxidative stress has been repeatedly proposed as a contributor to Tourette syndrome pathophysiology, and convergent neuroimaging evidence is emerging. In particular, magnetic resonance spectroscopy studies have reported brain biochemical changes consistent with oxidative stress in Tourette syndrome, supporting the plausibility that peripheral redox-related perturbations may coexist with central oxidative vulnerability [22]. Although serum metabolomics does not directly read out brain glutathione turnover, glutathione pathway enrichment is compatible with systemic oxidative or inflammatory signaling that may interact with neuroimmune crosstalk.

The concurrent alteration of glutamate and glycine provides an additional mechanistic bridge to excitatory–inhibitory balance within cortico-striatal circuits. Glutamate is the principal excitatory neurotransmitter and is recycled through astrocytic metabolism, while glycine is an inhibitory neurotransmitter in selected circuits and acts as a co-agonist at NMDA receptors, thereby modulating glutamatergic tone. Peripheral changes in glutamate, glycine, lactate, and glycerol reflect systemic metabolic adaptation and may influence, but do not directly represent, synaptic or circuit-level concentrations; however, consistent pathway signals across plasma metabolomics studies and meta-analytic biomarker synthesis support the relevance of glutamatergic and amino-acid metabolism in Tourette syndrome biology [19,20].

Alterations in lactate and glycerol add an energetic component consistent with stress-responsive neurobiology. Lactate reflects systemic glycolytic flux and is also a circulating substrate transported into the brain; within the brain, astrocyte–neuron metabolic cooperation uses lactate as both a fuel and a signaling molecule, capable of influencing neuronal activity and redox state [23]. Glycerol is largely generated through adipose triglyceride hydrolysis and is handled predominantly by the liver for gluconeogenesis and lipid synthesis, thereby linking adipose–liver energy trafficking with circulating metabolic tone. Such energetic shifts are biologically plausible in neurodevelopmental disorders characterized by heightened arousal and stress reactivity, where sympathetic activation and inflammatory signaling can reprogram peripheral substrate utilization.

Previous studies using proton magnetic resonance spectroscopy (MRS) have reported altered neurometabolic profiles in the brains of individuals with Tourette syndrome. For example, DeVito et al. observed reduced levels of neuronal markers such as N-acetylaspartate (NAA), creatine, and myo-inositol in the basal ganglia and frontal cortex, suggesting compromised neuronal integrity within cortico-striato-thalamo-cortical circuits [24]. More recent work has highlighted cerebral signatures consistent with oxidative stress, supporting the notion that redox dysregulation may contribute to TS pathophysiology [23]. While direct MRS studies in PANS are lacking, structural and diffusion MRI evidence indicate alterations in cortico-basal ganglia circuitry, which may reflect neuroinflammatory processes similar to those proposed in TS [25].

Our findings of altered serum metabolites, including arginine, proline, glutamate, and glycine, as well as energetic intermediates lactate and glycerol, complement these central observations. Although peripheral metabolite levels do not directly mirror synaptic concentrations, the convergence of peripheral and central markers suggests that systemic metabolic adaptations and neuroimmune interactions may influence cortico-striatal circuit function. These observations reinforce the biological plausibility of the metabolomic alterations detected in TS and their potential relevance for understanding convergent mechanisms in TS and PANS. All these findings should be interpreted with caution, as serum metabolomic profiles do not directly reflect brain-specific biochemical processes. Peripheral metabolic alterations may represent systemic physiological or immune responses rather than direct markers of central nervous system dysfunction. Therefore, any mechanistic link between the observed metabolic changes and neural circuit alterations remains indirect and requires further validation.

It is also important to underline that the absence of metabolomic separation between TS and PANS may reflect shared biology, limited statistical power, clinical heterogeneity, age or treatment effects, and/or the sensitivity limits of ^1^H NMR spectroscopy. However, the results suggest that, within the limits of the measured serum metabolome, both conditions share upstream drivers converging on amino acid, nitrogen, and redox metabolism. This interpretation is consistent with prior ^1^H NMR studies in PANS reporting perturbations in amino acid pathways and oxidative-stress-related signatures, including glutamate-related pathways and glutathione-associated processes [15,16].

Even though the etiopathogenesis is complex and multifactorial, immune dysfunction and chronic neuroinflammation have recently been regarded as pivotal causal pathways of TS [7]. Therefore, it does not unexpected that convergent metabolomic pathways between TS and PANS emerged in our research.

Strong evidence suggests that an immunogenetic factor may contribute to TS symptoms.

For instance, a study on a single-nucleotide polymorphism (SNP) in the TNF gene (-308 A/G), which codes for the proinflammatory cytokine tumor necrosis factor (TNF-α), has been associated with TS [26].

Also, a lymphocytic gene set driven by variants in the FLT3 gene has been described in TS as a factor leading to a neuroinflammatory determinant of the disorder [27]. According to these data, GWAS studies show a strong association between immune/inflammatory and allergic disorders and TS, as well as with other psychiatric disorders [28]. Furthermore, maternal and family history of autoimmune/inflammatory conditions (e.g., celiac disease, psoriasis, thyroiditis, and type 1 diabetes mellitus) is very frequent in subjects with TS [29]. These data alone would be sufficient to recall etiopathogenetic commonality between TS and PANS. But if this is not enough, an atypical activation of the innate immune response to bacterial components has also been described in TS and PANS. For instance, a lower expression of Toll-like receptor 4 (TLR4) after stimulation with lipopolysaccharides mimicking bacterial infections, and higher levels of soluble Cluster of Differentiation (CD) 14, has been demonstrated in a TS sample [30]. Moreover, it has been noted for some time that TS individuals have a lack of peripheral regulatory T cells (Tregs) and an overactivated immune response compared with neurotypical subjects [31]. Some authors have argued that, in some individuals with TS, an Immunoglobulin (Ig) subclass deficiency (e.g., IgG3) may hinder the removal of intracellular pathogens, reducing complement activation and leading to systemic and brain chronic inflammation [29,32]. Finally, serum anti-D2R antibodies have been found in subjects with TS, and their variable levels have been called upon to explain the fluctuating exacerbations of TS [33].

Since similar mechanisms have been identified in the genesis of PANS, it is not at all surprising that the two disorders also have a coincident metabolomic profile. On the other hand, the PANS phenotype comprises symptoms that largely overlap with those of all other NDDs, including TS, outlining a spectrum of disorders that share common pathogenetic pathways [34].

## 5. Limitations

Several limitations warrant emphasis. Serum metabolites integrate contributions from multiple organ systems and are sensitive to diet, fasting status, circadian rhythms, physical activity, and pharmacotherapy; future studies should standardize pre-analytical conditions and use more harmonized controls. The lack of separation of metabolomic profiles could also reflect limited statistical power for between-disease contrasts, reinforcing the need for larger, harmonized cohorts with symptom-state annotation, infection timing, and medication exposure. Although our results suggest convergent immunometabolic pathways, limitations include small cohort size, cross-sectional design, and diet and circadian influences, highlighting the need for cautious interpretation and validation in larger, phenotyped cohorts. We also point out that the average age of the control subjects is slightly higher than that of the clinical subjects, but the difference is so small that it does not constitute a real bias. Finally, ^1^H NMR spectroscopy offers high reproducibility but limited sensitivity for low-abundance metabolites, and complementary mass spectrometry and multi-omics integration will be important for mechanistic resolution.

## 6. Conclusions

This ^1^H NMR serum metabolomics study identifies a biochemical fingerprint associated with Tourette syndrome that centers on amino acid and nitrogen metabolism (arginine, proline, glutamate, and glycine), energetic intermediates (lactate and glycerol), and pathway enrichment consistent with altered urea-cycle and ammonia-recycling flux and glutathione-related redox regulation.

The absence of measurable separation between Tourette syndrome and Pediatric Acute-Onset Neuropsychiatric Syndrome supports substantial overlap in systemic biochemical perturbations, plausibly reflecting convergent neuroimmune activation and downstream metabolic reprogramming that interacts with cortico-striatal circuit function.

To date, few studies have investigated the distinction between Tourette syndrome and PANS to identify specific diagnostic markers, and we believe our study, although preliminary and exploratory, serves as a relevant starting point. Validation in larger, more deeply phenotypic cohorts, correlation with tic severity and comorbid features, and integration of longitudinal designs with immune profiling, microbiome characterization, and complementary analytical platforms will be required to determine whether this metabolomic fingerprint can support diagnostic workflows, risk stratification, or treatment monitoring.

## Figures and Tables

**Table 1:** Demographic data of the enrolled patients.

Classes	*N*	Female/Male	Age				
			Mean Value	Median	Mode	SD	Range
PANS	34	10/24	9.1	9	10	2.90	5–16
TS	23	3/20	9.9	10	10	2.75	4–15
Controls	25	9/16	12	11	11	2.17	8–17

Footnote: PANS = Pediatric Acute-Onset Neuropsychiatric Syndrome; TS = Tourette syndrome; SD = Standard deviation.

**Table 2:** Clinical parameters of PANS and TS groups.

PSYCHODIAGNOSTIC SCALES	PANS			Tourette		
	Mean Value	SD	Range	Mean Value	SD	Range
PANSS	47.24	19.94	14–100	33.30	12.86	10–55
YGTSS	33.29	26.22	0–87	31.13	15.94	0–65
CY-BOCS	10.12	8.54	0–26	2.96	5.61	0–19
PARS	11.47	8.79	0–30	8.48	6.77	0–22
TIQ	95.09	17.23	55–132	96.39	22.41	55–149
USCRS	6.44	9.10	0–38	3	7.60	0–33
C-GAS	50.79	14.06	24–80	55.13	17.17	32–81

Footnote: TS = Tourette syndrome; SD = Standard deviation; PANSS = Pediatric Acute Neuropsychiatric Symptom Scale; YGTSS = Yale Global Tic Severity Scale; CY-BOCS = Children’s Yale-Brown Obsessive Compulsive Scale; PARS = Pediatric Anxiety Rating Scale; TIQ = Total Intelligence Quotient (measured using WISC-IV = Wechsler Intelligence Scale for Children); USCRS = UFMG Sydenham’s Chorea Rating Scale; C-GAS = Children’s Global Assessment Scale.

**Table 3:** Statistical parameters of the multivariate analysis and their respective permutation tests.

Multivariate Analysis					
Models	R^2^X	R^2^Y	Q^2^	*p*-Value	Permutation Test: Intercept R^2^/Q^2^
Tourette vs. Control	0.54	0.42	0.4	0.02	0.27/−0.28
PANS vs. Tourette	0.36	0.31	−0.21	-	-
PLS Correlation Analysis					
*PSYCHODIAGNOSTIC SCALES*	R^2^				
PANSS	0.59				
YGTSS	0.65				
CY-BOCS	0.60				
PARS	0.58				
TIQ	0.60				
USCRS	0.62				
C-GAS	0.62				

PANS = Pediatric Acute-Onset Neuropsychiatric Syndrome; R^2^X, R^2^Y, and Q^2^ = variance and predictive ability established to evaluate the strength of the models; *p*-value = probability value.

## Data Availability

The original contributions presented in this study are included in the article. Further inquiries can be directed to the corresponding author.
